# Searching for General Model of Conspiracy Theories and Its Implication for Public Health Policy: Analysis of the Impacts of Political, Psychological, Structural Factors on Conspiracy Beliefs about the COVID-19 Pandemic

**DOI:** 10.3390/ijerph18010266

**Published:** 2020-12-31

**Authors:** Seoyong Kim, Sunhee Kim

**Affiliations:** 1 Department of Public Administration, Ajou University, Suwon 16499, Korea; seoyongkim@ajou.ac.kr; 2 Department of Local Government Administration, Gangneung-Wonju National University, Gangneung-si 25457, Korea

**Keywords:** general model of conspiracy theories, conspiracy theory, COVID-19 pandemic, social construction of conspiracy theory, belief in conspiracy theories

## Abstract

Along with the spread of the COVID-19 pandemic, beliefs in conspiracy theories are spreading within and across countries. This study aims to analyze predictors of beliefs in conspiracy theories. Because previous studies have emphasized only specific political, psychological, or structural factors or variables, this study constructs an integrated analytical model that includes all three factors. We analyze data from a large-scale survey of Koreans (*N* = 1525) and find several results. First, political, psychological, and structural factors influence beliefs in conspiracy theories. Second, when we examine the specific influences of the variables, we find that authoritarianism, support for minority parties, religiosity, trust in SNS (social networking services), perceived risk, anxiety, negative emotions, blame attribution, the quantity of information, health status, and health after COVID-19, all positively influence beliefs in conspiracy theories. Conversely, support for President Moon Jae-In’s government, Christianity, trust in the government, perceived control, analytic thinking, knowledge, the quality of information, and gender, all negatively impact these beliefs. Among the predictors, the quality of information, health status, support for President Moon Jae-In’s government, perceived risk, and anxiety have the most decisive impacts on beliefs in conspiracy theories.

## 1. Introduction

As COVID-19 spreads, conspiracy theories are spreading internationally and within certain countries. Example conspiracies include that COVID-19 is part of a government bioweapons program, that 5G cell towers are spreading COVID-19, and that pharmaceutical companies are encouraging the spread of COVID-19 for profit [1,2]. Many conspiracy theories existed before COVID-19. For example, conspiracy theories contended that President Obama was not born in the U.S., that Sandy Hook was a hoax, that the George W. Bush Administration knew about the 9/11 plot before it happened, and that John F. Kennedy was assassinated by the Central Intelligence Agency [3]. In the U.S., national opinion polls show that nearly 90% of Americans think that Lee Harvey Oswald did not act alone in killing John F. Kennedy [4]. Stempel et al. [5] reported that nearly one-third of American respondents accepted the theory that federal officials either facilitated the September 11th attacks or did nothing to stop them so that they could wage war in the Middle East. In Korea, prior to the outbreak of COVID-19, there existed political conflicts between conservative and progressive sectors. Such conflicts have manifested as various conspiracy theories after the outbreak of COVID-19. For example, on Pen and Mike TV, a YouTuber channel, a conservative YouTuber (Jeong Gyu-jae) said, “I have doubts whether Corona 19 is actually a dangerous disease [6].” “There are 300 deaths, being less serious than cold, influenza, and pneumonia, with more than 2000 deaths. Corona 19 is only a disease of the level of a seasonal cold, and the government is using it politically [3].” Other conspiracy rumors about COVID-19 in Korea have included: (1) that a person can get infected by going somewhere (e.g., a medical institution, restaurant, etc.) where a confirmed patient had visited, (2) that eating Chinese kimchi can cause infection, (3) that a mother and daughter who were confirmed to have been infected on a trip to Jeju Island were the family of the Vice Minister of the Ministry of Public Administration and Security, (4) that a necklace that generates chlorine dioxide is effective for preventing COVID-19, and (5) that the government recommends that medical staff wear regular gowns rather than full-body protective clothes. The Korean government clarified that this fake information was not true [7]. Previously, such conspiracy theories were thought to be believed by smaller groups of people, but more recently they have been gaining attraction with the general public. Thus, Miller et al. [3] argue that most people believe at least one conspiracy theory; conspiracy theories are not solely the domain of extremists and paranoid individuals. Based on nationally representative surveys, Oliver and Wood [8] show that most Americans consistently believe some conspiratorial story about a current political event or phenomenon.

People are thought to believe conspiracy theories for several reasons. According to Douglas et al. [9], conspiracy theories help to satisfy individuals’ social-psychological motives, including epistemic (understanding one’s environment), existential (feeling safe and in control), and social (maintaining positive images of one’s self and group) motives. Related to the COVID-19 pandemic, Earnshaw et al. [2] explain that conspiracy theories satisfy people’s existential motives by helping them to feel safe in their environments. Furthermore, Miller [3] views the increase in conspiracy theories in contemporary Western culture as a result of diminishing faith in governments and argues that this process may be exacerbated by new forms of media, such as the Internet.

Understanding that conspiracy theories are necessary for several reasons. Douglas et al. [10] argue that better understanding conspiracy theories is important because they have been closely linked to prejudice, witch hunts, revolutions, and genocide across history. Conspiracy theories can foster political extremism [11]. Also, believing one conspiracy theory is linked to beliefs in other conspiracy theories. Furthermore, in the field of health behavior, Earnshaw et al. [12] suggest that beliefs in conspiracy theories undermine engagement in pro-health behaviors and support for public health policies. During the 2014 Ebola epidemic, individuals who believed conspiracy theories about Ebola expressed less support for quarantine policies [12]. Many African Americans had higher levels of conspiracy beliefs about the origin and treatment of HIV/AIDS; these beliefs were related to distrust in treatment. Stronger beliefs in conspiracies contribute to health disparities by discouraging appropriate treatment behavior and play a role in declining vaccination rates [13]. Thus, conspiracy theories have negative effects in that they cause social distrust. However, they have some positive functions as well. According to Douglas et al. [10], conspiracy theories may empower individuals to challenge dominant hierarchies and question the behavior of powerful groups.

Understanding the spread of conspiracy theories related to COVID-19 is very important for preventing the disease’s spread and facilitating future treatment and vaccination processes. Thus, this study analyzes the drivers of beliefs in conspiracy theories. Previous studies in the fields of politics, psychology, and sociology have analyzed these drivers as well. Because the existing research models have not been integrated, however, there are limitations in fully explaining previous results. Thus, this study constructs more general model from integrated perspective. This study examines how political, psychological, and social structural factors, which consists of 21 variables, impact on beliefs in conspiracy theories related to COVID-19.

## 2. Theory and Hypotheses

### 2.1. Three Approaches to Conspiracy Theories

Conspiracy theories abound in social and political discourse, but a coordinated research agenda to grapple with their causes and consequences has been developed only in the last decade ([1,2,4,5,10,12,14], p. 3). Thus, diverse definitions of conspiracy theories have been put forth. For example, a conspiracy theory can be described as “*a subset of false beliefs in which the ultimate cause of an event is believed to be due to a plot by multiple actors working together with a clear goal in mind, often unlawfully and in secret*” [14]. Alternatively, a conspiracy theory can be defined as a belief that an event, situation, or set of people are controlled by unknown or secret forces, which usually have unsavory intentions ([15], p. 103). Finally, a conspiracy theory can be thought of as a “*proposed explanation of events that cites as a main causal factor a small group of persons (the conspirators) acting in secret for their own benefit, against the common good*” ([16], p. 2).

Some common features of conspiracy theories are that they supposedly let ordinary people in on secrets that the elite have tried to hide and that uncovering conspiracies can help to explain phenomena that were previously difficult to understand ([15], p. 104). Thus, Douglas et al. [10] regard conspiracy theories as attempts to explain the ultimate causes of significant social and political events and circumstances through claims of secret plots by two or more powerful actors. Moreover, conspiracy theories revolve around events that cause great confusion. For example, many people have believed the conspiracy theories that the U.S. government conspired in the 9/11 terrorist attacks [17] and that the assassination of President John F. Kennedy was not carried out by Lee Harvey Oswald alone [4]. Moreover, conspiracy theories develop because they offer individuals a way to interpret information that is difficult to organize or understand. Also, conspiracy theories are difficult to disprove because the counterarguments against them can be seen as parts of other conspiracy theories.

Analyses of the drivers of beliefs in conspiracy theories emphasize different factors depending on the academic discipline. Stempel et al. [5] categorized research on conspiracy theories into psychological and social structural approaches. Douglas et al. [7,8] list political, psychological, and social structural factors as the three main factors influencing beliefs in conspiracy theories.

First, from a political perspective, political power, ideology, and party politics can explain conspiracy theories. According to Douglas et al. [10], political scientists focus on how conspiracy theories become part of political contests, what political factors lead to conspiracy beliefs, and when conspiracy theories are mobilized as persuasive political tools. Moreover, political scientists focus on the roles of political factors, such as political partisanship and ideological self-identification, which are significantly associated with people’s beliefs in conspiracy theories [18]. For example, Enders and Steven [18] show that political orientation and conspiratorial thinking provide the most analytical leverage in predicting individuals’ beliefs in conspiracies. They argue that political orientation is a better predictor of beliefs in conspiracy theories than other attitudes, predispositions, and orientations. Moreover, Stempel et al. [5] demonstrate that beliefs in conspiracies are linked with mainstream political party divisions, and they argue that conspiracy thinking is a normal part of mainstream political conflict in the U.S. However, not every aspect of conspiracy theories can be explained solely by political factors. In addition to partisanship, individual differences in ways of interpreting information are related to beliefs in conspiracy theories [16].

Second, studies that focus on psychological factors pay attention to people’s psychological content and ways of thinking. According to Stempel et al. ([15], p. 354), a more psychological approach supports the existence of a conspiratorial personality or paranoid style of thought and views conspiracy theories as closely related to scapegoating and “us versus them” worldviews. In addition, Goertzel [4] puts forth the concept of a monological belief system in which similar beliefs comprise a self-sealing and expanding network of ideas that mutually support each other. Some conspiracy beliefs are correlated with each other as part of a monological belief system. Psychologists stress the psychological antecedents of conspiracy beliefs and have studied motivation, cognition, and personality as influencing factors. For example, from a psychological perspective, conspiracy theories help to satisfy people’s motivations. Douglas et al. [12] explain that these social-psychological motives include (1) the epistemic desire for understanding, accuracy, and subjective certainty; (2) the existential motive for control and security; and (3) the social motive to maintain a positive image of the self or group. In addition, the psychological approach emphasizes cognitive aspects of conspiracy theories. Miller [19] argues that conspiracy theories essentially play two cognitive roles: the argumentative and social critique roles. Moreover, Swami et al. [17] show that beliefs in conspiracies related to 9/11 are positively related to the Big Five personality factor, e.g., agreeableness.

The psychological approach maintains consistency between psychological elements, which is not observed in practice. Thus, Douglas et al. [10] argue that conspiracy theories are not always mutually supportive; instead, they often directly contradict one another. In addition, Uscinski and Parent [20] note that explanations of conspiracy theories that stress psychological needs are incomplete; although many stimuli cause stress, not all of them increase beliefs in conspiracies.

Third, structural explanations focus on social or contextual factors that constrain individuals. Stempel et al. [5] view social structural approaches as emphasizing the social structuring of beliefs and the social relativistic bracketing of conspiracy theories’ truth claims. The social structural approach focuses on race, social class, resources, and knowledge, which can limit individual behavior, as factors that influence beliefs in conspiracy theories. Crocker et al. [21] show that race is a significant predictor of beliefs in conspiracies even when controlling for other socioeconomic variables. Blaming the system is a much stronger predictor of conspiracy beliefs for Black students than for White students. In addition, Stempel et al. [5] demonstrate that less powerful social groups, such as racial minorities, lower social classes, women, and younger people, are more likely to believe in conspiracies. However, the structural approach overlooks the political reality and the influence of individuals’ independent psychological thinking.

In short, it is necessary to consider political, social, and structural factors together to explain conspiracy theories. It is about matter of general model for conspiracy theories. Some studies attempting to take an integrated approach have recently emerged. Swami et al. [17] show that beliefs in 9/11 conspiracies are positively related to exposure to 9/11 conspiracist ideas, beliefs in other conspiracy theories, defiance of authority, political cynicism, and the Big Five personality factor of agreeableness. This study focuses on 19 hypotheses related to beliefs in conspiracy theories, and the variables based on these hypotheses can be classified as political, psychological, or structural factors.

### 2.2. Political Factors

#### 2.2.1. Authoritarianism

Authoritarianism as a political attitude is characterized by preferences for conventionalism, authoritarian aggression, and authoritarian submission to authority [22]. Several empirical studies find that authoritarianism has a consistent relationship with beliefs in conspiracy theories. Authoritarian ideologies are characterized by unshakable beliefs in their righteousness, which provide instruments to comprehensively explain complex events [23]. Abalakina-Paap et al. [24] demonstrate that high levels of authoritarianism are related to beliefs in specific conspiracies. Swami [25] shows that right-wing authoritarianism (a measure of support for traditional social norms and submission to authority) is associated with stronger beliefs in general conspiracy theories. Swami et al. [17] show that beliefs in 9/11 conspiracy theories are positively related to defiance of authority. Moreover, based on a nationally representative sample of the U.S. population from the 2016 American National Election Studies, Goldberg and Richey [26] show that three different beliefs in conspiracies are positively correlated with authoritarianism. However, Oliver and Wood [8] do not find beliefs in conspiracies to be the product of greater authoritarianism, ignorance, or political conservatism.

**Hypothesis** **1 (H1).** *Authoritarianism is positively related to beliefs in conspiracy theories.*

#### 2.2.2. Ideology

Gruzd and Mai [27] find that although much of the content of conspiracy theories starts from users with limited reach, the initial propellants of these conspiracies are prominent conservative politicians and far-right political activists on Twitter.

The relationship between conspiracy theories and ideology has several explanations. People believe in conspiracy theories that fit their ideologies. Miller et al. [3] argue that those who endorse conspiracy theories have particular ideological worldviews with which the conspiracy theories can be associated. Miller et al. [3] show that left-right political orientation influences beliefs in conspiracy theories about climate change, Barack Obama’s birthplace, the 9/11 terror attacks, and electoral fraud. Additionally, believing in a conspiracy theory is highly likely when one’s belief in a specific ideology is strong [10]. Van Prooijen et al. [28] focus on political extremism. They show that extreme left and right political ideologies are positively associated with tendency to believe conspiracy theories. In the other hand, members of ideological groups tend to think that those who hold one ideology regard the other side’s argument as a conspiracy theory [10]. Hart and Graether [29] find a negative association between liberalism and beliefs in conspiracy theories. However, Oliver and Wood [6] demonstrate that beliefs in conspiracy theories are not limited to just one side of the ideological spectrum.

**Hypothesis** **2 (H2).** *Conservatives exhibit more beliefs in conspiracy theories than progressives do.*

#### 2.2.3. Partisanship

Political interests and competitions result in partisanship, which can manifest as party identification, involvement, and membership. These factors can influence beliefs in conspiracy theories. For example, party identification is associated with the endorsement of conspiracy theories that make the rival party look bad (e.g., Berinsky [30]). Enders and Smallpage [31] conduct an experimental study showing that conservative Republicans appear to be more susceptible to conspiratorial cues than progressive Democrats. When informational cues recede, conspiracy beliefs significantly increase among Republicans, even when a Republican is implicated by the conspiracy theory. However, Smallpage et al. [32] argue that it is false assumption that certain parties tend to believe in conspiracy theories more than other parties; instead, parties’ beliefs vary depending on the content of the conspiracy theory. The degree of belief in a particular conspiracy theory depends on political interests. For example, in the U.S., Democrats believe conspiracy theories about Republicans and conservatives, whereas Republicans believe conspiracies about Democrats. Gruzd and Mai [27] demonstrate that in late March 2020, hashtags containing fake news in favor of President Trump were being circulated and highlighted by Trump supporters. In the other and, the goal of party politics is to win an election. Thus, losers rather than winners tend to believe conspiracy theories. Uscinski and Parent [18] demonstrate that those that strongly endorse conspiracy theories are much more likely to be affiliated with the party in power.

**Hypothesis** **3 (H3).** *Support for parties in power (i.e., President Moon Jae-In’s government (Moon’s government, hereafter) and the Democratic Party in Korea) is negatively related to beliefs in conspiracy theories.*

#### 2.2.4. Religion

Religion greatly influences individual values and attitudes. Bezalel ([33], p. 1) argues that the nature of religious belief is important in understanding the epistemological foundations of worldviews that support conspiracy theories amidst what may be called conspiratorial ambiguity. Douglas et al. [10] and Frank et al. [34] explain that cognitive processes that are linked to conspiracy beliefs are connected to the acceptance of quasi-religious mentalities.

Both religion type and religiosity affect beliefs in conspiracy theories. Religiosity is the depth of faith in religion. Higher religiosity can be associated with greater beliefs in conspiracy theories because religions and conspiracy theories share certain elements, including paranormal activity, esotericism, millennialism, and prophecy [35]. Various interpretations are possible for the relationship between religious beliefs and conspiracy theory beliefs. For example, according to Jasinskaja-Lahti and Jetten [36], there is not difference of the belief in conspiracy theories between believers and non-believers. In other hand, they reported that the extent to which religious worldviews were endorsed predicted belief in conspiracy theories; not self-categorization as religious, but strong attachment to religion among believers was directly related with higher belief in conspiracy theories. Why strong attachment brings out more belief in conspiracy theory. Jasinskaja-Lahti and Jetten [36] explained this by the mediated role of higher anti-intellectualism which strong religious believers have. Individuals who show more orientation in scientifically sceptical attitude are more sensitive on find out logical fallacies presented in conspiracy theories [37]. Other studies reported that there is closely link between religiosity and stronger conservatism and traditionalism [38], and higher political conservatism [39]. Those conservatism induces more belief in conspiracy theories. Van der Linden et al. [40] showed that extreme conservatives were significantly more likely to engage in conspiratorial thinking than extreme liberals. Hart and Graether [29] show that believers in conspiracies tend to be more religious. In contrast, Jasinskaja-Lahti and Jetten [36] show that religious believers and non-believers do not differ in terms of beliefs in conspiracy theories.

Beliefs in conspiracies vary with the type of religion. For example, Marchlewska et al. [41] demonstrate that Catholic collective narcissism predicts not only outgroup hostility but also gender conspiracy beliefs. It is worth noting that the opinion on Catholics cannot apply to all Christians. Relevant [42] explains that Christians should forgo the temptation to look toward conspiracy theories to feel more secure or in control.

**Hypothesis** **4 (H4-1).** *Religiosity is positively associated with beliefs in conspiracy theories.*

**Hypothesis** **4 (H4-2).** *Different types of religions have different impacts on beliefs in conspiracy theories.*

#### 2.2.5. Trust

Distrust plays a fundamental role in the spread of beliefs in conspiracy theories. Miller et al. [3] argue that those who strongly think conspiracy theories believe that the world is a place where secretive, malevolent actions are not only possible but also probable. Thus, people with low trust levels have stronger beliefs in conspiracy theories. Miller et al. [3] show that trust is negatively associated with believing rumors. According to Hart and Graether [29], people who find it difficult to trust others may find solace in worldviews that blame hidden villains for disappointing outcomes.

Many empirical studies focus on the impact of trust on beliefs in conspiracy theories. Abalakina-Paap et al. [24] show that low levels of trust and hostility are related to attitudes regarding the existence of conspiracies in general. Distrust in a targeted object, such as politics, the government, or medicine, leads to beliefs in conspiracy theories. Governments have always been at the center of conspiracy theories. Georgiou et al. [30] show that beliefs in conspiracies are correlated with more negative attitudes toward government’s responses. In politics, conspiracies have always been hidden under the veil of power conflicts. Goldberg and Richey [26] find that three different beliefs in conspiracies are negatively correlated with political trust. Swami et al. [17] show that beliefs in conspiracies about 9/11 are positively related to political cynicism. Moreover, Einstein and Glick [43] find that political scandals decrease trust in the government, which, in turn, is linked with stronger beliefs in conspiracies. In addition, according to Earnshaw et al. [10], medical conspiracy beliefs are partly rooted in medical mistrust or a general suspicion of and lack of confidence in medical organizations and providers. According to Jin et al. [44], respondents who do not believe conspiracies trust information about COVID-19 from their doctors more than information from other sources, including social media. However, conspiracies are spread not only by trust but also in the presence of ignorance, conflict, and power. Miller et al. [3] show that a combination of high knowledge and low trust yields the greatest endorsement of conservative conspiracy theories among conservatives.

**Hypothesis** **5 (H5).** *Trust is negatively associated with beliefs in conspiracy theories.*

### 2.3. Psychological Factors

#### 2.3.1. Perceived Risk

In conspiracy theory research, beliefs in conspiracy theories are occurred under uncertainty and randomness which are related to social crises and risks [45,46]. Societal crisis situations increase beliefs in conspiracy theories because the unpleasant feelings that people experience when in crisis—fear, uncertainty, and a lack of control—motivate them to make sense of the situation, increasing the likelihood that they perceive conspiracies in social situations [41]. Uncontrollable danger from a personal perspective reinforces beliefs in conspiracy theories. Based on a dangerous worldview scale, Hart and Graether [29] show that people who report greater beliefs in conspiracy theories tend to hold more dangerous world beliefs.

Believing a conspiracy theory gives an individual a sense of security by lowering uncertainty. Earnshaw et al. [2] explain that conspiracy theories satisfy an existential motive by helping people feel safe in their environments; individuals are more likely to believe conspiracy theories when they feel anxious, powerless, and unable to control their outcomes. Similarly, people who view the world as dangerous and uncontrollable may assuage their anxiety through conspiracy theories [29].

**Hypothesis** **6 (H6).** *Perceived risk is positively related to beliefs in conspiracy theories.*

#### 2.3.2. Anxiety

From a psychological perspective, anxiety and stress should be lowered. Believing a conspiracy theory is one way of lowering them. Anxiety may be particularly acute if it is caused by a major external event, which may be a natural disaster or a human-caused event, such as a terror attack [30]. Conspiracy theories functionally provide very simple causal explanations for distressful events. In other words, they help to control the level of acute stress and, thus, instill order, a sense of control, and predictability [47]. High-anxiety situations are therefore positively correlated with conspiracy theories about Jewish people, Germans, and Arabs [48]. Moreover, based on experimental studies in which participants were exposed to mock news articles, Radnitz and Underwood [49] show that an anxiety prime increases beliefs in conspiracy theories. However, according to Swami et al. [47], state and trait anxiety and episodic tension are not significant predictors of beliefs in conspiracy theories.

**Hypothesis** **7 (H7).** *Anxiety is positively related to beliefs in conspiracy theories.*

#### 2.3.3. Negative Emotions

According to Whitson et al. ([50], p. 89), emotions that reflect uncertainty about the world (e.g., worry, surprise, fear, or hope) activate the need to imbue the world with order and structure across a wide range of compensatory measures, such as believing conspiracy theories, more so than other emotions (e.g., anger, happiness, disgust, or contentment) do. Van Prooijen et al. [51] propose that conspiracy theories are emotional; negative emotions rather than rational deliberations cause conspiracy beliefs. This insight is based on the argument that unpleasant emotional experiences increase sense-making motivations [52].

Several empirical studies support the emotional attributes of beliefs in conspiracy theories. Butler et al. [53] examine the emotional effects of viewing the film JFK on moviegoing audiences and find that it impacts viewers’ emotions, beliefs, and judgments, particularly regarding the themes and persuasive message of the film. However, the film does not influence viewers’ general political judgments or perceptions of conspiracies in their lives. Whitson et al. [54] demonstrate that experiencing uncertain emotions causes people to embrace conspiracies.

**Hypothesis** **8 (H8).** *Negative emotions are positively related to beliefs in conspiracy theories.*

#### 2.3.4. Perceived Control

Perceived control refers to an individual’s perception of his or her ability to control events and the extent of external objects [54]. Believing conspiracy theories is a way to create a sense of control. Beliefs in conspiracy theories are widely considered to be a product of a perceived lack of control [55]. Miller et al. [3] argue that those with strong beliefs in conspiracy theories are able to see how endorsing the conspiracy can serve their own stakes. Douglas et al. [10] explain that people who lack control may seek a sense of control by believing conspiracy theories because such theories provide an opportunity to refuse official narratives and allow people to feel that they have a better understanding. High levels of powerlessness, along with low levels of self-esteem, are related to beliefs in specific conspiracies, whereas high external locus of control levels, along with low trust levels, are associated with endorsements of conspiracies [24]. A sense of lacking control may cause people to adopt conspiracy-like thinking. Sullivan et al. [56] use an experiment to demonstrate that participants with no control over given topics increase their endorsement of specific conspiracy theories. The result shows that the feeling of control over COVID-19 is very low, leading to conspiracy theories. Earnshaw et al. [2] argue that the COVID-19 pandemic provides a powerful context for people to utilize conspiracy theories in an attempt to restore feelings of safety and control.

However, based on six studies conducted online using MTurk samples, Stojanov et al. [55] show that changes in levels of control have no effect on conspiracy theory beliefs. Thus, conspiracy beliefs are not suitable compensation for threats to control. Similarly, Hart and Graether [29] find no effects of situational threats (i.e., a sense of powerlessness) on beliefs in conspiracy theories.

**Hypothesis** **9 (H9).** *Perceived control is negatively associated with beliefs in conspiracy theories.*

#### 2.3.5. Analytic Thinking

Thinking style plays an important role in this context because beliefs in conspiracy theories depend on human judgment. Many studies investigate the connections between analytic and heuristic thinking and beliefs in conspiracy theories. For example, Leman and Cinnirella [57] conducted an experiment in which participants are asked to read stories about the president’s assassination and then rate the likelihood of different explanations to measure analytic or heuristic thinking. They report that the participants are likely to endorse a conspiracy theory to account for events more when the consequences are major (i.e., the president dies) than when they are comparatively minor. In addition, people who are less likely to engage in analytic thinking [37] or more likely to rely on heuristics [58] tend to rely on conspiracy theories. Beliefs in conspiracy theories are positively related to intuitive rather than analytic thinking [37].

**Hypothesis** **10 (H10).** *Analytic thinking is negatively associated with beliefs in conspiracy theories.*

#### 2.3.6. Blame Attribution

When socially negative events occur, the responsible targets to be blamed are often up for debate. Blame attribution is closely related to conspiracy theory belief structures. Clark [59] argues that conspiracy theories are a byproduct of a fundamental attribution error. Hart and Graether ([29], p. 230) explain that conspiracy worldviews provide consolation for individuals who have difficulty seeing reality through a more benevolent lens. For such individuals, the belief that someone is responsible for negative events may be preferable to concluding that the universe is cruel and unjust. Thus, conspiracy theories are associated with aspects of justice and responsibility, one of which is related to blame attribution.

In an empirical study, Crocker et al. [19] demonstrate that beliefs in conspiracy theories are related to blaming the problems of Black Americans on prejudice and discrimination. Moreover, this race effect is partially mediated by a measure of system blame but not by the greater externality of attributional style. Schulzke [60] shows that attributional uncertainty facilitates the assignment of blame to familiar enemies, which ultimately promotes conspiratorial thinking.

**Hypothesis** **11 (H11).** *External blame attribution is positively related to beliefs in conspiracy theories.*

### 2.4. Structural Factors

#### 2.4.1. Social Class

Uscinski and Parent [20] argue that conspiracy theories exist for “losers.” Thus, socially underprivileged individuals and members of the lower class tend to believe conspiracy theories. Mao et al. [61] show that social class can significantly negatively predict individuals’ beliefs in conspiracy theories and explain the likely link between social class and individuals’ beliefs. Because individuals from lower social classes receive less education, live in harsher environments, and often face external threats, they feel less control over their external environments. Generally, when perceived control is threatened by external factors, beliefs in conspiracy theories increase. Van Prooijen [62] finds that social class is correlated with the extent to which individuals believe conspiracy theories.

A representative indicator of social class is income. In empirical studies, Golec de Zavala and Federico [63] show that higher income reduces beliefs in conspiracy theories. Furthermore, Uscinski and Parent [20] demonstrate that people who work in the financial industry or for the government or the military exhibit the lowest levels of conspiracy thinking. However, the causal relationship between conspiracy beliefs and income is indeterminate [10].

**Hypothesis** **12 (H12).** *Belonging to a higher social class (i.e., having a higher income in this study) is negatively related to beliefs in conspiracy theories.*

#### 2.4.2. Knowledge

Knowledge plays critical roles in reducing beliefs in conspiracy theories. Miller et al. [3] show that those who are highly knowledgeable about politics tend to be the most susceptible to conspiracy theories. Moreover, this knowledge mediates the effect of ideology on the endorsement of conspiracy theories; knowledge increases ideologically motivated endorsements of conspiracy theories among conservatives. Moreover, Berinsky [31] shows that more knowledgeable people are less likely to believe in political conspiracies than their low-knowledge counterparts are. Goldberg and Richey [26] demonstrate that three different beliefs in conspiracy theories are positively correlated with political knowledge.

**Hypothesis** **13 (H13).** *Knowledge has negative impact on beliefs in conspiracy theories.*

#### 2.4.3. Education

Many studies examine the relationship between education and conspiracy theories. Generally, lower education levels increase beliefs in conspiracy theories [7,61,64]. Van Prooijen [62] suggests that education may give people a set of cognitive and affective attributes that enable them to resist conspiracy theories. She describes the causal chain from education to beliefs in conspiracy theories as follows: less education → less analytic thinking → strong beliefs in simple solutions → greater beliefs in conspiracy theories. Also, Georgiou et al. [30] show that beliefs in conspiracy theories related to COVID-19 are greater among people with lower levels of education.

**Hypothesis** **14 (H14).** *Education is negatively associated with beliefs in conspiracy theories.*

#### 2.4.4. Information

Information performs a function similar to that of knowledge. But both the quantity and quality of information are related to beliefs in conspiracy theories. In terms of the quantity, information related to a conspiracy theory reinforces beliefs in that theory. For example, Swami et al. [17] show that beliefs in conspiracies about 9/11 are positively related to exposure to the ideas of 9/11 conspiracists. In terms of quality, more elaborate information decreases beliefs in conspiracies. For example, more news media literacy is shown to reduce conspiracy theory endorsement [65].

It is not only the quantity and quality of information but also the style of information processing that influences beliefs in conspiracy theories. A conspiratorial mentality may partly reflect particular information-processing dispositions [29]. Thus, individuals’ engagement in seeking or finding meanings or patterns in ambiguous or random information may be related to conspiratorial thinking.

**Hypothesis** **15 (H15).** *More qualitative and quantitative information is negatively associated with beliefs in conspiracy theories.*

#### 2.4.5. Social Support

Social structures that shape citizens’ feelings of vulnerability increase beliefs in conspiracy theories [66]. As a social structure, a social network or social support acts as a shield against conspiracy theories. Conversely, a lack of social resources or support promotes the beliefs in conspiracy theories. Freeman and Bentall [67] show that conspiracy believers are more likely to have weaker social networks. According to Grohol [68], any societal alienation appears to be connected to greater beliefs in conspiracy theories. Those who suffer on the edge of society, because of their unemployment status, minor ethnicity, or even weak relationship status, report stronger beliefs in the conspiracy theories. Conversely, Sapountzis and Condor [69] find that conspiracy narratives are as likely to be used by people with large social networks as by those whose social interactions are generally more restricted.

**Hypothesis** **16 (H16).** *Individuals with more social support exhibit weaker beliefs in conspiracy theories.*

#### 2.4.6. Health Status

In the movie *Conspiracy Theory*, the main character, portrayed by Mel Gibson, is a patient who believes in a conspiracy theory. Even in the real world, health has a significant impact on beliefs in conspiracy theories. Barron et al. [70] note that the trait of schizotypy, characterized by perceptual, cognitive, and affective abnormalities, has been found to be a strong, positive predictor of beliefs in conspiracy theories. Coltheart [71] suggests that false beliefs occur because of neuropsychological impairments that (1) enforce the formation of such beliefs owing to faulty sensory information and (2) make it difficult to reject such beliefs owing to faulty prefrontal systems that evaluate thoughts. In addition, March and Springer [72] use a regression model to show that apart from odd beliefs or magical thinking and Machiavellianism, primary psychopathy is a significant positive predictor of beliefs in conspiracy theories. Conversely, Chen et al. [73] provide evidence that beliefs in conspiracy theories regarding the COVID-19 virus can predict the mental health and well-being of healthcare workers. Good health creates positive emotions and, consequently, weakens beliefs in conspiracy theories. Thus, the following hypothesis can be established.

**Hypothesis** **17 (H17).** *Better health status is negatively associated with beliefs in conspiracy theories.*

#### 2.4.7. Age/Gender

Among demographic variables, age and gender have been examined as predictors of beliefs in conspiracy theories. According to Radnitz et al. [49] and Swami [25], young people are more likely to believe in conspiracy theories than older people. Moreover, in Goreis and Voracek’s [74] metastudies, youth is a positive predictor of a beliefs in conspiracy theories. However, Earnshaw et al. [2] find no significant impact of age on conspiracy beliefs. 

Regarding gender, Radnitz et al. [47] show that men have stronger beliefs in conspiracy theories than women have. Hart and Graether [29] show that conspiracy believers tend to be younger and female. Cassese et al. [75] demonstrate that men believe conspiracy theories more than women do because the former have higher levels of personal uncertainty and learned hopelessness than the latter have. In contrast, however, March and Springer [72] find that gender and age do not statistically significantly affect beliefs in conspiracy theories.

**Hypothesis** **18 (H18).** *Younger people exhibit stronger beliefs in conspiracy theories than older people do.*

**Hypothesis** **19 (H19).** *Women exhibit stronger beliefs in conspiracy theories than men do.*

## 3. Sample and Measures

This study analyzes survey data (*N* = 1,525) collected from a sample of people in Korea from 6 August 2020, to 11 August 2020. Korea Research, a survey research institute, conducted an online survey for seven days to collect the data. Korea Research’s online panel comprises 460,000 candidate survey respondents. First, an e-mail was sent to 9839 panelists. Of them, 2083 opened the e-mail, and 1,525 ultimately finished the survey. To obtain a representative sample of the Korean population, the survey used a quota sampling method that considered the proportions of respondents by region, gender, and age. A sampling error of ±2.5% at the 95% confidence level was adopted, assuming random sampling.

Of the respondents, 47.9% are male and 52.1% are female. By age, 16.7% of respondents are 18 to 29 years old, 16.3% are 30 to 39 years old, 19.6% are 40 to 49 years old, 20.3% are 50 to 59 years old, and 27.1% are 60 or older (*N* = 414). By educational level, 47.2% of the respondents are high school graduates or below, whereas 52.8% (*N* = 805) attended and graduated from college. Finally, in terms of monthly household income, 15.5% earn less than 2 million won, 17.2% earn between 2 and 3 million won, 21.4% earn between 3 and 4. 1 million won, 16.5% earn between 4 to 5 million won, 10.5% earn between 5 to 6 million won, 7.5% earn between 6 to 7 million won, and 11.4% earn 7 million won or more.

Beliefs in conspiracy theories are measured by seven items. We developed these items based on previous studies on conspiracy theories [1,2]. The seven questions are structured to include politicians, governments, countries, and pharmaceutical companies, which are the subjects of relevant conspiracies. The specific items are as follows. (1) Politicians do not honestly reveal their true intentions to the public regarding their decisions on coronavirus disease (COVID-19). (2) There is a secret organization that greatly influences political decisions. (3) The government is hiding something from the public. (4) The government is always monitoring the public. (5) The government makes important decisions related to coronavirus disease (COVID-19) without the public knowing. (6) Certain powerful nations deliberately created the coronavirus (COVID-19) to dominate the world. (7) Coronavirus disease (COVID-19) was deliberately created by pharmaceutical companies to make money.

Table 1 shows the content and reliability of the measurement items for each independent variable. Most of the responses for each question are measured on a five-point Likert scale. Ideology is measured on a ten-point scale, with higher scores indicating progressiveness and lower scores indicating conservativeness. Partisanship is measured through support for the current government and support for political parties. In Korea, President Moon Jae-in is in power, so the degree of support for the Moon government is measured. In addition, the respondents chose the political party that they supported. This question had six possible answers: ① Democratic Party (ruling party), ② Integration Party (a major opposition party), ③ Justice Party, ④ People Party, ⑤ other parties, and ⑥ no party supported. For religion, the survey included measures of both religiosity and type of religion. The type of religion was classified as Catholic, Christian, Buddhist, or non-religious. Depending on the target, trust was measured as trust in the government, doctors, SNS (social networking services), or people. Both quantitative and qualitative aspects of information were measured. For health status, both overall health status and the degree of deterioration of health after COVID-19 were measured.

## 4. Analysis and Findings

### 4.1. Descriptive Analysis

**Figure 1:**
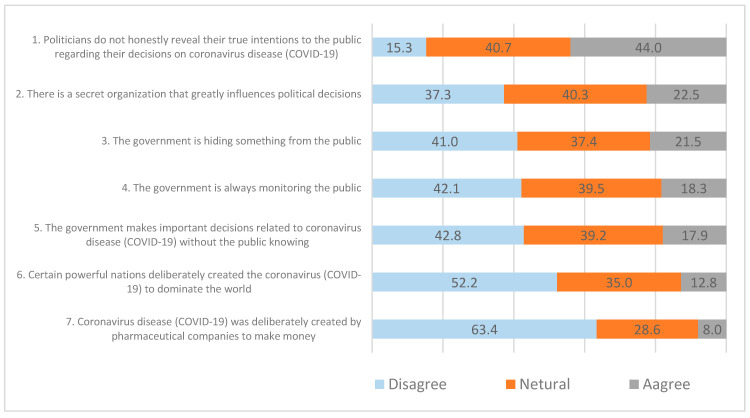
Frequency of beliefs in conspiracy theories.

A simple frequency analysis of the items measuring conspiracy theories is shown in Figure 1. The statement “Politicians do not honestly reveal their true intentions to the public regarding their decisions on coronavirus disease (COVID-19)” has the most support. This result suggests that political distrust plays an important role in the spread of conspiracy theories because politicians are included in that measure. The second item with a high agreement rate is “there is a secret organization that greatly influences political decisions.” This result also indicates the influence of politics in the spread of conspiracy theories because this item concerns politics as well. Next, 18.3% and 17.9% of respondents support “the government is hiding something from the public,” and “the government is always monitoring the public.” Both statements are related to the government. However, 41.0% and 42.1% of respondents disagree with these statements, which implies that most people do not believe government-related conspiracy theories. Additionally, 12.8% of respondents agree with “certain powerful nations deliberately created the coronavirus (COVID-19) to dominate the world,” which suggests the existence of conspiracies at the international level. However, 52.2% of respondents disagree with this statement. In addition, only 8.0% agree and 63.5% disagree with the statement “coronavirus disease (COVID-19) was deliberately created by pharmaceutical companies to make money.”

The simple frequency analysis shows, first, that although a fairly large number of respondents disagree with conspiracy theories, some people do believe them. Second, the degree of belief in a conspiracy theory depends on the main subject of the theory. For example, respondents are most likely to believe conspiracy theories related to politicians and least likely to believe conspiracy theories related to doctors. Third, a significant proportion of respondents expresses neutral attitudes, that is, “neither agree nor disagree.” The highest proportion of neutral responses is 40.7%, and the lowest is 28.6%. The findings implies that many people may believe in conspiracy theories, even if they are explicitly expressed.

**Figure 2:**
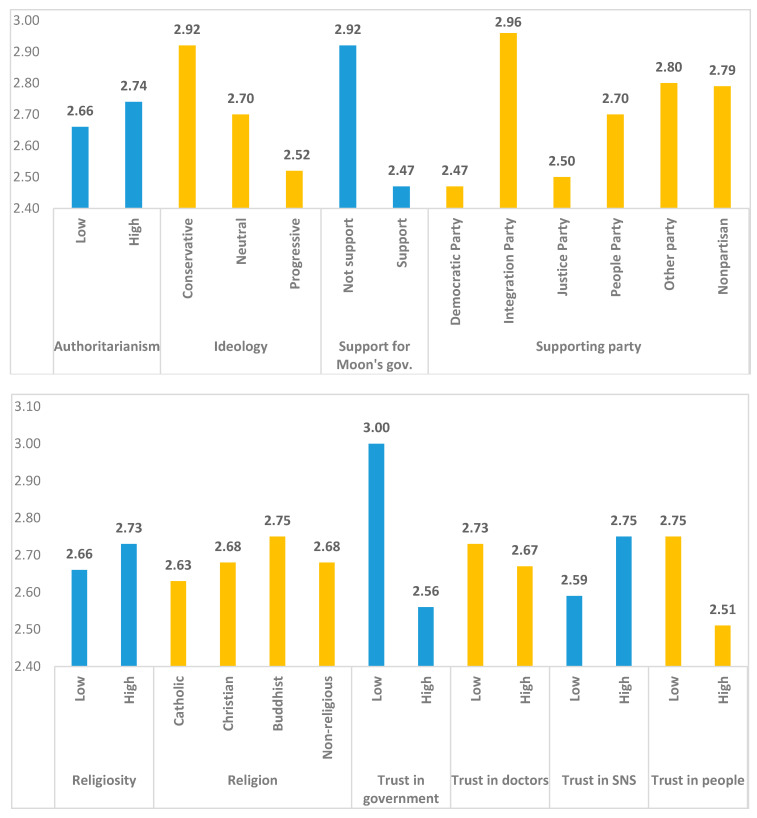

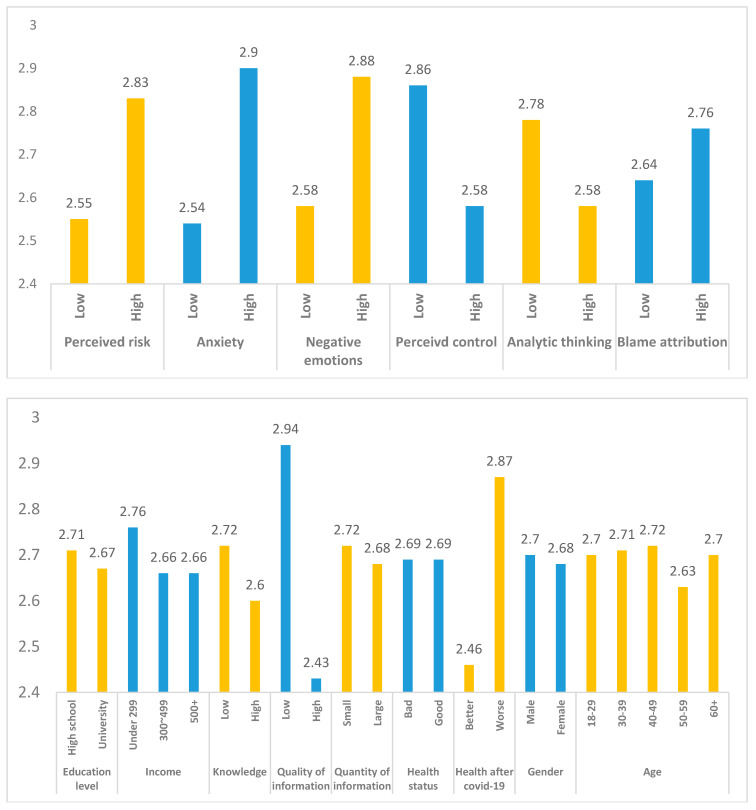
Means by groups in predictors.

Differences in beliefs in conspiracy theories are analyzed across different groups as shows in Figure 2. Excluding the categorical variables, we divide the respondents into two or more groups based on the average values of items measured on a five-point scale. Generally, the higher group includes respondents with scores above the average value, whereas the lower group includes respondents with scores below the average value.

First, in terms of political factors, the group with high authoritarianism scores has stronger beliefs in conspiracy theories than the group with low authoritarianism scores, and this difference is statistically significant (*F*-value = 4.533, *p*-value = 0.033). Ideologically, conservatives have stronger beliefs in conspiracy theories than progressives do, which supports the results of Hart and Graether [29]; this difference is also statistically significant (*F*-value = 14.635, *p*-value = 0.000). The degree of belief in conspiracy theories varies depending on whether the respondent supports Moon’s government. Beliefs in conspiracy theories are stronger among the group that does not support Moon’s current government (*F*-value = 150.362, *p*-value = 0.000). These results confirm those of Uscinski and Parent [20]. Additionally, supporters of the current ruling Democratic Party do not tend to believe conspiracy theories (*F*-value = 17.904, *p*-value = 0.000). The group with a higher degree of religiosity indicates stronger beliefs in conspiracy theories than the lower group does (*F*-value = 3.511, *p*-value = 0.061). Buddhists tend to have the strongest beliefs in conspiracy theories, followed by Christians, non-religious people, and Catholics. Buddhists may have stronger beliefs in conspiracies because the majority of them are conservative elderly. However, the differences between these groups are not statistically significant (*F*-value = 1.086, *p* = 0.354).

Among the trust variables, individuals with lower trust in the government exhibit stronger beliefs in conspiracy theories than those with higher trust (*F*-value = 124.413, *p*-value = 0.000). However, trust in doctors is not significantly associated with beliefs in conspiracy theories (*F*-value = 2.207, *p*-value = 0.138). The group with higher trust in SNS has stronger beliefs in conspiracy theories than the group with low trust (*F*-value = 15.019, *p*-value = 0.000). Higher trust in the general public is significantly associated with weaker beliefs in conspiracy theories (*F*-value = 31.972, *p*-value = 0.000). Among the four trust groups, the classification based on trust in the government has the largest difference between the low and high groups. The impact of trust in the government therefore seems to be very large.

When the groups are defined according to psychological variables, all of the differences are statistically significant. First, the group with high risk perception has a higher level of trust in conspiracy theories than the group with low risk perception (*F*-value = 92.182, *p*-value = 0.000). The groups with higher anxiety (*F*-value = 92.182, *p*-value = 0.000) and negative emotions (*F*-value = 61.314, *p*-value = 0.000) also have stronger beliefs in conspiracy theories than the corresponding lower groups. The groups with lower perceived control (*F*-value = 52.502, *p*-value = 0.000) and analytic thinking (*F*-value = 28.828, *p*-value = 0.000) have stronger beliefs in conspiracy theories than the corresponding higher groups. Those who exhibit more external blame attribution by assigning responsibility for problems to others, express stronger beliefs in conspiracy theories (*F*-value = 9.49, *p*-value = 0.002).

Among the structural factors, lower education levels are associated with stronger beliefs in conspiracy theories, but the difference is not statistically significant (*F*-value = 0.922, *p*-value = 0.337). Beliefs in conspiracy theories are high among households with incomes below 300 million won and are relatively lower in the two groups with incomes of 300 million won or more (*F*-value = 3.368, *p*-value = 0.035). The more knowledgeable the respondents are and the better their quality of information is, the lower their beliefs in conspiracy theories (knowledge, *F*-value = 7.905, *p*-value = 0.005; quality of information; *F*-value = 211.000, *p*-value = 0.000). In particular, the wide gap in groups with the high and low information suggests that the quality of information is very important. Notably, differences in the amount of information are not associated with any difference in conspiracy beliefs (*F*-value = 0.916, *p*-value = 0.339). This result suggests that the quality of information is more important than the quantity of information. Health status has no significant effect (*F*-value = 0.027, *p*-value = 0.870), whereas the change in health status (worse) after COVID-19 does have a significant effect (*F*-value = 127.523, *p*-value = 0.000). These results show that health changes according to variations in context are more important than everyday health status. Finally, neither gender nor age has a statistically significant impact (gender, *F*-value = 0.422, *p*-value = 0.516; age, *F*-value = 0.695, *p*-value = 0.596).

### 4.2. Correlation Analysis

We use Pearson’s simple correlations to examine the simple relationships between variables, and the results are shown in Table 2. Categorical variables, such as religion type and party type, are excluded from this analysis. For variables that are measured on a five-point Likert scale, we take the average values of multiple measures to create composite variables.

First, we investigate the relationship between belief in conspiracy theories and political factors. Conspiracy beliefs are positively related to authoritarianism, religiosity, and trust in SNS, whereas they are negatively related to ideology, support for Moon’s government, trust in the government, and trust in people. Authoritarianism and religion have no statistically significant relationships.

From a theoretical point of view, the finding that progressives do not believe conspiracy theories supports the results of previous studies [29]. The fact that trust in conspiracy theories is not high when support for Moon’s government is strong implies that conspiracy theories are more popular among political losers. The correlation coefficient of trust in doctors is interesting; Trust in doctors has no significant relationship with conspiracy theories. This finding is unexpected, as doctors play an important role in COVID-19.

Beliefs in conspiracy theories depend on the type of trust. Trust in the government and trust in the general public weaken conspiracy beliefs, whereas trust in SNS strengthens them. It can be inferred that conspiracy theories spread online and that online trust is contrary to the offline trust structure. Correlation coefficients of trust in the government is the largest, indicating the importance of the government’s role in handling the COVID-19 pandemic. Among the political variables, support for Moon’s government and trust in the government have large correlation coefficients, which also indicate that the government plays a significant role in a pandemic.

Among the psychological factors, beliefs in conspiracy theories are positively related to perceived risk, anxiety, negative emotions, and blame attribution whereas are negatively related to perceived sense of control and analytic thinking. Perceived risk, anxiety, and emotions may be byproducts of a negative effect. Thus, it is important to reduce people’s negative and pessimistic thinking during the COVID-19 pandemic. In addition, it is noteworthy that negative emotions and analytic thinking have opposite relations with beliefs in conspiracy theories. This opposition demonstrates the typical contradictory roles of emotion and reason. In addition, analytic thinking and blame attribution are related to modes of thinking and logic. This result therefore suggests that it is necessary to perform an in-depth dissection of the general public’s mode of thinking under COVID-19. The psychological variable with the largest coefficient is negative emotions, suggesting the importance of emotional thinking during the COVID-19 pandemic.

Regarding structural factors, which mainly include sociodemographic variables, beliefs in conspiracy theories are positively related to health status after COVID-19 and are negatively related to income, knowledge, and the quality of information. The quality of information has the largest correlation, suggesting that high-quality information can help to reduce beliefs in conspiracy theories. The significant roles of both knowledge and information quality attest to the importance of literacy in enlightening the public. The fact that health status has no statistically significant correlation but a negative change in health status after COVID-19 has a significant correlation suggests that the change in health after COVID-19 is more important than the usual health status. The results that education level, gender, and age have no significant effects differ from previous findings [29,48].

Among all variables, the quality of information has the largest correlation coefficients, followed by trust in the government, support for Moon’s government, and negative emotions. These variables belong to the political, psychological, and structural factors, suggesting that beliefs in conspiracy theories depend on various factors rather than on one specific factor.

### 4.3. Regression Analysis

To examine the determinants of beliefs in conspiracy theories, we conduct a regression analysis with beliefs in conspiracy theories as the dependent variable and political, psychological, and structural factors as independent variables. Some of the independent variables are dummy variables. For these variables, the reference groups are the middle group in the cases of ideology, the non-partisan group in party supported, the non-religious group in religion, the group with income below 5 million won in income, the group with less than a college degree in education, and the male in gender, respectively. The regression analysis is carried out separately for each of the three factors, and the results are shown as Models 1, 2, and 3 in Table 3.

In Model 1, the political variables, authoritarianism, religiosity, and trust in SNS, positively influence beliefs in conspiracy theories. Conversely, support for the current the President Moon’s government, Christianity, trust in the government, and trust in people, all have negative effects. Ideology and partisan support do not have significant influences. For the religion variables, religiosity has a statistically significant effect whereas Christianity negatively influences beliefs in conspiracies. This suggests that not only the depth of religious belief but also the type of religion plays an important role in beliefs in conspiracy theories. In particular, it is noteworthy that the two variables play opposite roles in determining beliefs in conspiracy theories. Because some extreme Christians in Korean society disseminate conspiracy theories and oppose the current government, the result that general Christians do not believe conspiracy theories may indicate that there is a difference in conspiracy beliefs between general Christians and extreme believers. For the trust variables, the degree and direction of beliefs in conspiracy theories vary depending on the object of trust. Trust in the government and trust in people decrease beliefs in conspiracy theories, whereas trust in SNS increases them. The fact that trust in the government reduces beliefs in conspiracy theories suggests that citizens can accept the government’s active role in the response to COVID-19. In particular, the fact that trust in the government has the largest standardized regression coefficient among the trust variables suggests that the government certainly should play an active role in handling the COVID-19 pandemic. The fact that trust in SNS leads to stronger beliefs in conspiracy theories implies that that information related to conspiracy theories is common on SNS in the COVID-19 pandemic, which affects Internet users.

In Model 1, the coefficient of trust in the government is the largest based on standardized regression coefficient values, meaning that it has the most explanatory power, followed by support for the current government, trust in SNS, and authoritarianism. This result implies that the government’s role in the COVID-19 pandemic is important because the top two variables in terms of explanatory power are related to the government.

In Model 2, perceived risk, anxiety, negative emotions, and blame attribution have positive effects on beliefs in conspiracy theories, whereas perceived control has a negative effect. These results match the hypotheses. Analytic thinking negatively affects beliefs in conspiracy theories, but the relation is not statistically significant. They are all negative attributes, meaning that more larger efforts are needed to effectively decrease negative mood and perceptions in the COVID-19 pandemic. From this perspective, because an individual’s perceived sense of control is a psychological variable that plays a role in decreasing beliefs in conspiracy theories, it is necessary to strongly empower people to see the more positive sides of situations. In addition, beliefs in conspiracy theories are related to external blame attribution, in which people place responsibility for COVID-19 on other people rather than on themselves. External rather than internal blame attribution reinforces beliefs in conspiracy theories. This finding implies that an emphasis on individual responsibility in the course of the COVID-19 pandemic is important to enable people to attribute blame internally. Among the six variables in Model 2, the most influential variable is negative emotions, followed by anxiety, perceived control, and perceived risk. This result suggests that an emotional rather than a rational approach is needed to reduce beliefs in conspiracy theories during the COVID-19 pandemic.

Model 3 shows the impacts of the structural variables on beliefs in conspiracy theories. Knowledge and the quality of information positively affect these beliefs, whereas the amount of information, health status, and worsening health status after COVID-19 have negative impacts. Education level, income, gender, and age have no statistically significant effects. The first important point is about to the roles of education and knowledge. The former does not affect beliefs in conspiracy theories, whereas the latter does affect them. These results suggest that rather than increasing formal education, a knowledge-centered approach is needed to reduce beliefs in COVID-19 conspiracy theories. Second, the quality and quantity of information perform opposite functions. The former decreases beliefs in conspiracy theories, whereas the latter increases such beliefs. This result implies that the large quantities of information available on SNS contain conspiracy theories, suggesting that high-quality information is needed to address the large quantities of information about conspiracy theories. Third, the two health variables have opposite effects. Generally, health increases beliefs in conspiracy theories, but a deterioration in health after COVID-19 leads to stronger beliefs in conspiracy theories. The information quality variable has the greatest explanatory power in Model 3, followed by health status, the quantity of information, and health status after COVID-19. The results confirm the importance of information and health.

Model 4 shows the causal relationships when all variables are included in the regression. Most of the directions and statistical significance of the effects found by Models 1, 2, and 3 are maintained. However, there are a few differences. First, beliefs in conspiracy theories increase when respondents support other parties outside the mainstream. This result suggests that conspiracy theories may spread to minority or alternative parties rather than to mainstream or established parties. Second, trust in the general public, which has a significant effect in Model 1, has no significant effect when all variables are included. Third, analytic thinking, which has no significant effect in Model 2, now has a significant effect. This finding suggests that thinking patterns and logic can suppress beliefs in conspiracy theories. Fourth, it appears that women do not believe conspiracy theories to the extent that than men do. This finding supports existing research results [29].

Model 4 includes all variables that affect beliefs in conspiracy theories. The variables with the largest standardized regression coefficients are the quality of information (−0.260), health status (0.154), support for Moon’s government (−0.141), perceived risk (0.128), and anxiety (0.104). These results suggest that the quality of information is very important in suppressing beliefs in conspiracy theories. In addition, the five variables belong to each political, psychological, and structural components, which suggests that a balanced approach that considers multiple variables at the same time should be used to address conspiracy theories.

Finally, Model 4′s explanatory power for beliefs in conspiracy theories is 35.2%. Considering the large number of independent variables, its power is rather small. Thus, it is necessary to identify additional variables to increase the model’s explanatory power. The explanatory power of Model 1 is 20.8%, that of Model 2 is 17.3%, and that of Model 3 is 24.8%, suggesting that political, psychological, and structural factors are all important.

## 5. Conclusions

### 5.1. Findings and Summary

The main findings are as follows. First, the simple frequency analysis shows that the percentage of respondents who agree with conspiracy theories ranges from 8.0% to 44.0%. The belief rates are high for conspiracy theories concerning politicians or politics. It is also noteworthy that the proportion of people who moderately believe conspiracy theories is significant, ranging from 28.6% to 40.7%. This result suggests the potential for conspiracy theories to spread in Korean society. Second, the mean analysis shows that beliefs in conspiracy theories differ greatly between groups supporting and opposing the current government; groups trusting and not trusting the government; groups with high and low perceived risk, anxiety, and negative emotions; groups with and without better qualitative information; and groups with and without poor health generally and after COVID-19. Third, the correlation analysis shows that the quality of information has the greatest correlation with beliefs in conspiracy theories, followed by trust in the government, support for Moon’s government, and negative emotions. Fourth, the regression analysis shows that among the independent variables, authoritarianism, support for minority parties, religiosity, trust in SNS, perceived risk, anxiety, negative emotions, blame attribution, the quantity of information, health status, and health after COVID-19, all have positive effects on beliefs in conspiracy theories. Support for Moon’s government, Christianity, trust in the government, perceived control, analytic thinking, knowledge, and the quality of information and gender (female), all have negative effects. Overall, we consider 19 variables that influence beliefs in conspiracy theories. The finding that women have less probability for believing in conspiracy theories than man is a sensitive for interpretation. It is possible that men’s power orientation seems to be strengthen their belief in conspiracy theory. However, this assumption should also be proved through further research. The variable with the largest standardized regression coefficient is the quality of information, followed by health status, support for Moon’s government, perceived risk, and anxiety. These results show that the quality of information is very important in suppressing beliefs in conspiracy theories. The explanatory power of Model 1 is 20.8%, that of Model 2 is 17.3%, and that of Model 3 is 24.8%. This result confirms that all three factors are important for explaining beliefs in conspiracy theories and suggests that additional variables are needed to increase the model’s explanatory power.

### 5.2. Implications and Discussion

First, this study provides implications regarding which information variable has the greatest impact on beliefs in conspiracy theories. Specifically, such beliefs decrease as the quality of information improves. In addition, increasing the quantity of information spreads these beliefs. This finding suggests that communications with the public should include high-quality information rather than large amounts of information. In addition, because conspiracy theories are highly likely to be embedded in large quantities of information, it is necessary to filter or monitor information content.

Second, this study found that political, psychological, and structural factors simultaneously influence beliefs in conspiracy theories. This result suggests that these three factors should be considered in a balanced way to suppress conspiracy theories. Because the three factors have very different properties, various approaches are needed. A political approach should consider the structure of negotiations, the distribution of interests, and the competitive structure of winners and losers. A psychological approach should be mindful of the structure of thinking and the possibility for individuals to change. A structural approach should consider ways to change the social structure’s fundamental framework rather than individual minds.

Third, the simple frequency analysis results show that although the percentage of respondents who believe conspiracy theories is relatively small, many respondents take a more neutral position. Because this neutral group may become a potential resource for the spread of conspiracy theories, it is necessary to analyze its characteristics and prepare to decrease this group’s active role.

Fourth, within the political factor, authoritarianism, support for Moon’s government, support for other parties, religiosity, Christianity, trust in the government, and trust in SNS, all influence beliefs in conspiracy theories. Because ideological factors, such as authoritarianism and religiosity, are difficult to manipulate artificially, possible approaches to reduce conspiracy theories using these factors are limited. However, support for Moon’s government and trust in the government can be impacted through the intentional efforts by the government to secure trust. To build trust during the COVID-19 pandemic, the government must transparently disclose information, communicate with the public, and make strategic efforts to quickly respond to problems. In the context of COVID-19, studies have discussed ways to make good use of trusted actors. Gruzd and Mai [27] explain that the spread of misinformation can potentially be mitigated by fact-checking and directing people to credible information from public health agencies. Messages on inoculations from trusted opinion leaders can prevent beliefs in conspiracies and enhance intentions to be vaccinated [76]. Thus, Earnshaw et al. [2] suggest that increasing the uptake of COVID-19 vaccines when they become available and gaining support for COVID-19 public health-related policies require strategies that leverage trusted sources of COVID-19 information (e.g., doctors).

Fifth, within the psychological factor, all six variables significantly influence beliefs in conspiracy theories. In particular, the impacts of perceived risk and anxiety are large and significant. This result suggests that because the increase in anxiety during the COVID-19 pandemic can lead to the spread of conspiracy theories, efforts are needed to control anxiety. In particular, the fact that a sense of control reduces beliefs in conspiracy theories suggests that it is important to give individuals confidence that they can control COVID-19. The government should organize effective quarantine measures to control COVID-19 and actively promote them to the public. In addition, because perceived risk, anxiety, and negative emotions are all based on negative thinking, communication strategies must reinforce positive aspects of the situation. Beliefs in conspiracy theories decrease in the case of analytic thinking, implying a need for a policy that focuses on disseminating analytical information to the public. Additionally, because beliefs in conspiracy theories increase in the case of external blame attribution, it is necessary to emphasize individual responsibility in the COVID-19 pandemic.

### 5.3. Limitations and Further Research

This study attempted to analyze the determinants of beliefs in conspiracy theories by constructing a more integrated model. However, it has several limitations. First, the analysis focused on political, psychological, and structural factors, but these three factors cannot fully explain beliefs in COVID-19 conspiracy theories. As the explanatory power of the full model, including all three factors, is 35.2%, it is necessary to identify additional important factors and variables for each factor. In particular, this study overlooks not only a lot of perception and communication factors [77,78,79], but also structure ones [80,81,82,83,84,85,86] and cultural value ones [87,88,89,90,91,92]. Second, this study measures beliefs in conspiracy theories by focusing on conspiracy theories related to the COVID-19 pandemic. Thus, analytic approaches are needed to compare our findings with findings related to beliefs in other, more general conspiracy theories.

## Figures and Tables

**Table 1:** Variable measurement and reliability.

Variables	Measures	Scale	Reliability
Authoritarianism	A slightly dictatorial political leader is needed to resolve the coronavirus crisis.	Five-point scale (1. disagree, 5. agree)	0.806
	Control about free media is needed to respond to the coronavirus problem.		
	To solve the coronavirus problem, individual liberties must be suppressed to a considerable extent.		
Ideology	On a scale from one to ten points, where one point is the most conservative and ten points is the most progressive, where do you rate your political ideology?	Ten-point scale (1. conservative, 10. progressive)	-
Support for Moon’s government	How much do you support the Moon Jae-In administration?	Ten-point scale (1. disagree, 10. agree)	-
Religiosity	How much devoted to religion do you think you are?	Ten-point scale (1. not at all, 10. very much)	-
Trust in the government	How much do you trust the following subjects to provide coronavirus-related information? Government	Five-point scale (1. distrust, 5. trust)	-
Trust in doctors	How much do you trust the following subjects to provide coronavirus-related information? Doctors	Five-point scale (1. distrust, 5. trust)	-
Trust in SNS	How much do you trust the following subjects to provide coronavirus-related information? SNS	Five-point scale (1. distrust, 5. trust)	-
Trust in people	Generally speaking, how much do you think you can trust people in a relationship?	Five-point scale (1. can’t believe almost everyone, 5. can believe almost everyone)	-
Perceived risk	I am relatively more likely to get coronavirus compared to others	Five-point scale (1. disagree, 5. agree)	0.846
	I am more vulnerable to coronavirus compared to others		
Anxiety	After the coronavirus outbreak, how often do you feel the following items? 1. My nerves have become sensitive. 2. I have no hope. 3. I am anxious. 4. I am so depressed that nothing can comfort me. 5. I have no value or meaning. 6. I am worried. 7. I am depressed. 8. I am nervous. 9. I cannot concentrate. 10. I am lonely.	Five-point scale (1. very occasionally, 5. very often)	0.964
Negative emotions	I am annoyed when I come across coronavirus-related information.	Five-point scale (1. disagree, 5. agree)	0.719
	I feel anxious when checking coronavirus-related information.		
	I think the future is dark when I come across coronavirus-related information.		
Perceived control	The coronavirus problem can be overcome through human effort.	Five-point scale (1. disagree, 5. agree)	0.666
	Coronavirus-related risks can be overcome with my efforts.		
Analytic thinking	Rather than analyzing coronavirus-related information carefully and logically, I made judgments based on intuitive feelings.	Five-point scale (1. disagree, 5. agree)	0.816
	I interpret coronavirus-related information emotionally rather than rationally.		
Blame attribution	People other than me contributed more to the coronavirus outbreak.	Five-point scale (1. disagree, 5. agree)	0.700
	The responsibility for resolving the coronavirus outbreak lies with others rather than me.		
Knowledge	I am familiar with coronavirus disease.	Five-point scale (1. disagree, 5. agree)	0.840
	I know more about coronavirus disease than others do.		
Quality of information	Coronavirus-related information provided by the government is objective and based on facts.	Five-point scale (1. disagree, 5. agree)	0.912
	Coronavirus-related information provided by the government is scientifically based and professional.		
Quantity of information	I have more coronavirus-related information than others have.	Five-point scale (1. disagree, 5. agree)	0.889
	I have obtained a lot of meaningful information related to coronavirus disease.		
Health status	I am healthy.	Five-point scale (1. disagree, 5. agree)	0.901
	I am in good health compared to other people.		
Health after COVID-19 (worse)	My physical health deteriorated after COVID-19.	Five-point scale (1. disagree, 5. agree)	0.771
	My mental health deteriorated after COVID-19.		

**Table 2:** Pearson’s correlations among the study variables.

			1	2	3	4	5	6	7	8	9	10	11	12	13	14	15	16	17	18	19	20	21	22	23
1. Belief in the Conspiracy theories			1																						
Political Factor	2. Authoritarianism		0.040	1																					
	3. Ideology (progressive)		−0.161 ***	0.169 ***	1																				
	4. Support for Moon’s Gov.		−0.337 ***	0.258 ***	0.564 ***	1																			
	5. Religiosity		0.029	0.009	−0.006	0.066 *	1																		
	Trust	6. Trust in the government	−0.350 ***	0.217 ***	0.357 ***	0.565 ***	0.050	1																	
		7. Trust in doctors	−0.026	−0.023	−0.026	−0.028	0.084 **	0.128 ***	1																
		8. Trust in SNS	0.134 ***	0.051 *	−0.020	−0.020	0.162 ***	0.078 **	0.251 ***	1															
		9. Trust in people	−0.145 ***	−0.085 **	0.055 *	0.114 ***	0.088 **	0.123 ***	0.052 *	0.051 *	1														
Psychological factors	10. Perceived risk		0.235 ***	0.108 ***	0.020	−0.031	0.059 *	−0.036	0.055 *	0.096 ***	−0.078 **	1													
	11. Anxiety		0.294 ***	0.130 ***	0.019	−0.089 **	−0.037	−0.147 ***	−0.042	0.031	−0.206 ***	0.289 ***	1												
	12. Negative emotions		0.310 ***	0.138 ***	−0.011	−0.127 ***	0.030	−0.094 ***	0.067 **	0.184 ***	−0.093 ***	0.310 ***	0.361 ***	1											
	13. Perceived control		−0.193 ***	0.068 **	0.036	0.185 ***	0.078 **	0.287 ***	0.170 ***	0.113 ***	0.151 ***	−0.090 ***	−0.200 ***	−0.053 *	1										
	14. Analytic thinking		−0.134 ***	−0.202 ***	−0.077 **	−0.093 ***	−0.056 *	−0.120 ***	−0.041	−0.238 ***	0.023	−0.217 ***	−0.142 ***	−0.345 ***	−0.103 ***	1									
	15. Blame attribution		0.155 ***	0.148 ***	0.004	−0.042	−0.090 ***	−0.057 *	0.022	−0.025	−0.101 ***	0.035	0.155 ***	0.180 ***	−0.056 *	−0.039	1								
Structural factors	16. Education level		−0.024	−0.061 *	0.076 **	0.069 **	0.032	0.023	−0.008	−0.064 *	0.040	−0.061 *	−0.021	−0.068 **	0.036	0.073 **	0.014	1							
	17. Income		−0.051 *	−0.019	0.024	0.048	0.021	0.014	0.063 *	0.014	0.118 ***	−0.114 ***	−0.102 ***	−0.016	0.065 *	0.027	0.033	0.220 ***	1						
	18. Knowledge		−0.065 *	0.051 *	0.136 ***	0.123 ***	0.099 ***	0.121 ***	0.127 ***	0.116 ***	0.105 ***	0.075 **	0.001	0.050	0.235 ***	−0.045	0.084 **	0.084 **	0.054 *	1					
	19. Quality of information		−0.414 ***	0.215 ***	0.356 ***	0.582 ***	0.031	0.720 ***	0.125 ***	0.023	0.140 ***	−0.060 *	−0.166 ***	−0.099 ***	0.358 ***	−0.136 ***	−0.046	0.025	0.044	0.194 ***	1				
	20. Quantity of information		−0.033	0.151 ***	0.165 ***	0.247 ***	0.145 ***	0.290 ***	0.172 ***	0.208 ***	0.081 **	0.100 ***	0.024	0.145 ***	0.263 ***	−0.318 ***	0.012	0.042	0.034	0.454 ***	0.406 ***	1			
	21. Health status		0.013	0.020	0.075 **	0.083 **	0.058 *	0.123 ***	0.089 **	0.067 **	0.122 ***	−0.264 ***	−0.215 ***	−0.044	0.197 ***	−0.037	0.032	0.134 ***	0.185 ***	0.202 ***	0.162 ***	0.188 ***	1		
	22. Health status after COVID-19 (worse)		0.292 ***	0.100 ***	0.032	−0.106 ***	0.010	−0.146 ***	0.019	0.084 **	−0.094 ***	0.341 ***	0.444 ***	0.354 ***	−0.116 ***	−0.143 ***	0.132 ***	−0.036	−0.034	0.077 **	−0.183 ***	0.082 **	−0.146 ***	1	
	23. Gender		−0.017	0.038	0.059 *	0.000	0.072 **	−0.005	0.041	0.015	−0.049	−0.011	0.043	0.124 ***	−0.094 ***	0.023	0.031	−0.082 **	−0.027	−0.066 *	0.008	−0.045	−0.022	0.054 *	1
	24. Age		−0.010	0.027	−0.125 ***	−0.103 ***	0.231 ***	0.057 *	0.051 *	0.104 ***	0.076 **	0.104 ***	−0.171 ***	0.055 *	0.167 ***	−0.025	−0.156 ***	−0.154 ***	−0.088 **	0.041	0.023	0.065 *	−0.033	0.019	−0.003

Note: * *p* < 0.05; ** *p* < 0.01; *** *p* < 0.001.

**Table 3:** Multiple regression analysis findings.

			Model 1			Model 2			Model 3			Model 4		
			B	SE	Beta	B	SE	Beta	B	SE	Beta	B	SE	Beta
Constant			3.301	0.126		1.993	0.180		3.076	0.145		2.479	0.218	
Politi-cal factors	Authoritarianism		0.109 ***	0.020	0.137							0.055 ***	0.019	0.068
	Ideology	Conservative	0.044	0.051	0.022							−0.006	0.047	−0.003
		Progressive	−0.046	0.063	−0.019							0.021	0.058	0.009
	Support for Moon’s Gov.		−0.058 ***	0.009	−0.240							−0.034 ***	0.008	−0.141
	Supporting party	Democratic Party	0.024	0.050	0.016							0.019	0.046	0.012
		Integration Party	0.033	0.061	0.015							−0.007	0.057	−0.003
		Justice Party	−0.077	0.094	−0.020							−0.063	0.086	−0.016
		People Party	−0.001	0.130	0.000							−0.021	0.119	−0.004
		Other party	0.212	0.129	0.039							0.251 **	0.118	0.046
	Religion	Religiosity	0.024 ***	0.008	0.094							0.019 **	0.008	0.074
		Catholic	−0.089	0.064	−0.038							−0.073	0.059	−0.031
		Christian	−0.138 ***	0.060	−0.077							−0.096 *	0.055	−0.053
		Buddhist	−0.035	0.054	−0.018							−0.006	0.049	−0.003
	Trust	Trust in gov.	−0.176 ***	0.020	−0.256							−0.056 **	0.022	−0.081
		Trust in doctors	−0.022	0.019	−0.027							−0.017	0.018	−0.022
		Trust in SNS	0.115 ***	0.020	0.144							0.067 ***	0.019	0.084
		Trust in people	−0.083 ***	0.023	−0.087							−0.034	0.021	−0.036
Psychological factors	Perceived risk					0.094 ***	0.021	0.111				0.108 ***	0.021	0.128
	Anxiety					0.115 ***	0.020	0.147				0.081 ***	0.020	0.104
	Negative emotions					0.183 ***	0.027	0.187				0.097 ***	0.025	0.099
	Perceived control					−0.143 ***	0.024	−0.143				−0.045 *	0.024	−0.045
	Analytic thinking					−0.034	0.024	−0.036				−0.043 *	0.023	−0.046
	Blame attribution					0.078 ***	0.022	0.085				0.067 ***	0.021	0.072
Structural factors	Education level								−0.018	0.036	−0.012	0.026	0.034	0.017
	Income								−0.046	0.037	−0.028	−0.053	0.036	−0.033
	Knowledge								−0.095 ***	0.029	−0.084	−0.091 ***	0.028	−0.081
	Quality of information								−0.355 ***	0.021	−0.431	−0.214 ***	0.029	−0.260
	Quantity of information								0.136 ***	0.026	0.143	0.067 **	0.026	0.071
	Health status								0.101 ***	0.022	0.111	0.141 ***	0.021	0.154
	Health after COVID-19 (worse)								0.197 ***	0.021	0.224	0.070 ***	0.022	0.080
	Gender								−0.035	0.033	−0.023	−0.065 **	0.033	−0.044
	Age								−0.001	0.001	−0.013	0.000	0.001	−0.007
*F*-value			22.904 ***			52.769 ***			55.456 ***			24.824 ***		
R^2^/Adjusted R^2^			0.208/0.199			0.173/0.169			0.248/0.243			0.352/0.337		

Note: * *p* < 0.05; ** *p* < 0.01; *** *p* < 0.001.

## Data Availability

The data presented in this study are available on request from the corresponding author. The data are not publicly available due to regulations and guideline of data open policy according to the National Research Foundation of Korea.
